# Comprehensive care in cystic fibrosis: mental health assessments

**DOI:** 10.36416/1806-3756/e20250143

**Published:** 2025-05-21

**Authors:** Paulo Camargos

**Affiliations:** 1. Departamento de Pediatria, Faculdade de Medicina, Universidade Federal de Minas Gerais, Belo Horizonte (MG) Brasil.; 2. Unidade de Pneumologia Pediátrica, Hospital das Clínicas, Universidade Federal de Minas Gerais, Belo Horizonte (MG) Brasil.

People with chronic conditions, such as cystic fibrosis (CF), often experience mental health challenges. Effectively managing these complex diseases requires a comprehensive approach and a dedicated multidisciplinary care team, considering the correlation between distress levels and the severity of the condition.

CF is a chronic and potentially life-threatening condition that affects thousands of people in Brazil. Alongside its “organic” features, it is connected to various biopsychosocial issues that increase the risk of anxiety and depression for patients, as well as for their families and caregivers. Consequently, effective management of CF relies on a multidisciplinary team to promote overall wellness and address the challenges of managing this complex chronic condition. To support people with CF (pwCF) and their families, the team must be aware of, understand, and explore the multiple manifestations of the disease, including various clinical targets, to foster the best possible well-being for patients and their loved ones.

According to the Brazilian CF Patient Registry, the median survival age was 43 years between 1999 and 2017.^1^ ^,^ ^2^ In comparison, recent estimates indicate that North American CF patients born between 2019 and 2023 might have a median survival age of approximately 60 years.^3^ This suggests that as life expectancy for CF patients increases, caregivers and parents still face significant challenges. Effectively managing CF throughout life will improve the mental health of patients as their conditions evolve.

PwCF experience anxiety and depression based on validated assessments. These findings highlight the clinical relevance of the study by Rozov et al.^4^ published in this issue of the *Jornal Brasileiro de Pneumologia*: as survival rate increases, it becomes essential to incorporate mental health evaluations routinely-such as those proposed by the authors-into care protocols that have yet to be implemented in accordance with the recommendations of the Brazilian CF guidelines.^1^ Through the *Patient Health Questionnaire and Generalized Anxiety Scale,* the authors^4^ noted that their results demonstrate “*the importance of annual screening and suggest that tools for assessing depression and anxiety can be enormously useful for early detection, intervention, as well as for reducing stigma.”*

As we move into the era of CF transmembrane conductance regulator (CFTR) modulators, the significance of this care component increases. Integrating CFTR modulator drugs into clinical practice has yielded remarkable therapeutic results. These modulators have improved numerous patient outcomes by providing substantial benefits to those who qualify and can utilize them, decelerating disease progression and improving quality of life. Despite these positive outcomes, several side effects have been noted, such as a rise in psychiatric reactions following the start of using CFTR modulators in combination, transforming the psychosocial landscape of pwCF and including new challenges and psychosocial needs.

Many patients and their parents have reported experiencing negative neuropsychiatric side effects after starting combination therapy with CFTR modulators, adversely affecting their mental and psychosocial well-being. Commonly reported concerns include anxiety, depression, mood swings, and sleep disturbances, 60% of which classified as adverse events. Nonetheless, these effects remain poorly understood.^5^ Therefore, it is essential to include mental health assessments in routine care, regardless of the use of this medication class, and to integrate these assessments into the care protocol for patients undergoing these treatments. Thus, addressing psychiatric effects through proper evaluation, counseling, and ongoing monitoring are crucial. In other words, the findings establish assumptions for identifying future research priorities, guiding policy efforts, and enhancing communication in areas that promote the well-being of pwCF.

Caregivers and family members often devote countless hours to their caregiving roles and frequently report experiencing poor health. Consequently, enhancing dyadic coping could be beneficial. Interventions aimed at caregivers of pwCF could also improve home care management, bolster caregiver health, and foster better dyadic coping between caregivers and patients, ultimately alleviating the burden on caregivers. Therefore, developing a similar strategy to address the mental health needs of these individuals would be advantageous.

Additional regional or national studies utilizing the same tools reported and assessed by Rosov et al.,^4^ especially those with a sufficiently large and representative random sample, would deepen our understanding of the impact of CFTR modulators on the nervous system and CF itself by identifying opportunities and strategies to optimize psychosocial care.
